# Barriers and facilitators to maternal death surveillance and response at a busy urban National Referral Hospital in Uganda

**DOI:** 10.12688/openresafrica.13438.1

**Published:** 2022-09-14

**Authors:** Imelda Namagembe, Jolly Beyeza-Kashesya, Joseph Rujumba, Dan K.Kaye, Moses Mukuru, Noah Kiwanuka, Ashley Moffett, Annettee Nakimuli, Josaphat Byamugisha

**Affiliations:** 1 Department of Obstetrics and Gynaecology, School of Medicine, Makerere University College of Health Sciences, Uganda, P.O Box 7072, Kampala, Uganda, Makerere University and Mulago Specialized Women Neonatal Hospital, Kampala, Uganda, +256, Ugand, Makerere University and MSWNH, Kampala, Uganda, +256, Uganda; 2 Department of Obstetrics and Gynaecology, School of Medicine, Makerere University College of Health Sciences, Uganda, P.O Box 7072, Kampala, Uganda, Makerere University and Mulago Specialized Women Neonatal Hospital, Kampala, Uganda, +256, Ugand, Makerere University /MSWNH, Kampala, Uganda, +256, Uganda; 3 Department of Paediatrics and Child Health, School of Medicine, Makerere University College of Health Sciences, Uganda, P.O Box 7072, Kampala, Uganda, Makerere University, Kampala, Uganda, +256, Uganda; 4 Department of Obstetrics and Gynaecology, School of Medicine, Makerere University College of Health Sciences, Uganda, P.O Box 7072, Kampala, Uganda, Makerere University, Kampala, Uganda, +256, Uganda; 5 Department of Health Policy Planning and Management, School of Public Health, Makerere University College of Health Sciences, Uganda, P.O Box 7072, Kampala, Uganda, MakCHS, Kampala, Uganda, +256, Uganda; 6 Department of Epidemiology and Biostatistics, School of Public Health, Makerere University College of Health Sciences, Uganda, P.O Box 7072, Kampala, Uganda, MakCHS, Kampala, Uganda, +256, Uganda; 7 Department of Pathology and Centre for Trophoblast Research, University of Cambridge, Cambridge, United Kingdom, University of Cambridge, Cambridge, United Kingdom, +44, UK; 8 Department of Obstetrics and Gynaecology, School of Medicine, Makerere University College of Health Sciences, Uganda, P.O Box 7072, Kampala, Uganda, MakCHS, Kampala, Uganda, +256, Uganda; 9 Department of Obstetrics and Gynaecology, School of Medicine, Makerere University College of Health Sciences, Uganda, P.O Box 7072, Kampala, Uganda, Mak- CHS, Kampala, Uganda, +256, Uganda

**Keywords:** Key words: Barriers, facilitators, death reviews, multi-stakeholder perspectives

## Abstract

**Background: **Preventable maternal and newborn deaths remain a global concern, particularly in low- and middle-income countries. Timely maternal death surveillance and response (MDSR) is a recommended strategy to account for such deaths through identifying contextual factors that contributed to the deaths to inform recommendations to implement in order to reduce future deaths.

With the leadership of WHO and UNFPA, there is momentum to roll out MDSR, however, the barriers and enablers for implementation have received limited attention. These have important implications for successful implementation. The aim of this study was: To assess barriers and facilitators to implementation of MDSR at a busy urban National Referral Hospital as perceived by health workers, administrators, and other partners in Reproductive Health.

**Methods:** Qualitative study using in-depth interviews (24), 4 focus-group discussions with health workers, 15 key-informant interviews with health sector managers and implementing partners in Reproductive-Health. We conducted thematic analysis drawing on the Theory of Planned Behaviour (TPB).

**Results: **The major barriers to implementation of MDSR were: inadequate knowledge and skills; fear of blame and litigation; failure to implement recommendations; burn out because of workload at the National Referral Hospital and inadequate leadership- to support health workers. Major facilitators were involving all health workers in the MDSR process, eliminate blame, strengthen leadership, implement recommendations from MDSR and functionalize lower health facilities (especially Health Centre -IVs).

**Conclusions: **The barriers of MDSR include knowledge and skills gaps, fear of blame and litigation, and other health system factors such as erratic emergency supplies, and leadership/governance challenges. Efforts to strengthen MDSR for impact should use health system responsiveness approach to address the barriers identified, constructive participation of health workers to harness the facilitators and addressing the required legal framework.

## Introduction

Preventable maternal mortality has remained a global concern despite the 44% reduction that occurred from 2000 to 2015 ( UNICEF, 2019; UNFPA *et al.*, 2019). Uganda had a modest reduction in its maternal mortality ratio (MMR) over that millennium development goals (MDG) period, i.e. from 506 per 100,000 live births to 336 per 100,000 livebirths ( Uganda Bureau of Statistics and ICF, 2016). Overtime, women are dying from conditions considered preventable ( Kaye *et al.*, 2003; Nakimuli *et al.*, 2016; Ngonzi *et al.*, 2016). Implementation of a sustained maternal death surveillance response (MDSR) system is one of the innovations that is recommended as a strategy to reduce maternal deaths ( Koblinsky, 2017; World Health Organization, 2013; World Health Organization and UNICEF, 2015). Timely MDSR would enhance accountability at all levels of the health care system up to the community level in order to prevent maternal and newborn death ( Bandali *et al.*, 2016; Hunt & Gray, 2013; Koblinsky, 2017; Mathai *et al.*, 2015).

Indeed, implementation of recommendations from maternal death reviews and confidential enquiries has contributed to reduction in maternal deaths in some settings such as Rwanda ( Sayinzoga *et al.*, 2016), the United Kingdom ( Knight *et al.*, 2016), Ethiopia ( Abebe *et al.*, 2017; Lindtjørn *et al.*, 2017). However, the recommended timely notification and review of these deaths ( World Health Organization, 2021) does not occur in many parts of the world especially in Sub-Saharan Africa (SSA) where most of the maternal deaths occur ( Mathai *et al.*, 2015; Smith *et al.*, 2017a; Smith *et al.*, 2017b). There is especially limited information on barriers and facilitators to timely MDSR the high-volume settings in SSA where MDSR uptake is still low.

Drawing on the Theory of Planned Behaviour (TPB) ( Ajzen, 1991) we explored barriers and facilitators to strengthen MDSR system as a quality improvement process at a busy tertiary hospital in Uganda. The constructs within the theory have been reported to predict attitudes and intention to implement particular behaviour including health sciences ( Ajzen, 2015; Cooke *et al.*, 2016; McEachan *et al.*, 2011). In addition, the TPB has been used to understand behaviour and successfully applied in other studies including health sciences ( Bosnjak *et al.*, 2020; Wiese *et al.*, 2021). TPB has the following domains: Attitude; subject norms; perceived behavioral control; intention (plan to change to embrace a particular behavior or action, in this case MDSR); then planned behavior to effect or perform the actual practice. The TPB is widely used to explain behaviour in terms of the beliefs that individuals hold about the behaviour in question in this case MDSR.

## Methods

### Study setting

This work has been conducted in the Department of Obstetrics -Gynaecology of a high-volume National Referral Hospital. It was originally located on Mulago Hill (5 km North of Kampala City), but currently at Kawempe 7km North of Kampala City of Uganda. It is part of the wider research project whose title is *“Reducing maternal deaths using maternal death surveillance and response at Mulago-Kawempe National Referral Hospital in Uganda **”.***

### Study participants

Most of the study participants (internal stakeholders) work at the Hospital which also doubles as the main teaching hospital for Makerere University and other medical training institutions. These included: obstetricians, midwives, senior house officers (resident medical officers on masters training program), representatives from laboratory, pharmacy, stores, anesthesia providers, administrators and hospital managers. These participants were mainly from labour ward, theatre, High-Dependency Unit (HDU) and gynae-emergency where most of the maternal deaths occur.

The other participants (external stakeholders) were representatives from Reproductive Health division of Ministry of Health (MoH), implementing partners in Reproductive Health such as WHO, UNFPA, Kampala Slum Maternal and Newborn (MaNe) Project, regulatory body for medical doctors (Uganda Medical and Dental Practioners’ Council -UMDPC) and representative from Uganda Medical Association. The external stake holders were people familiar with the MDSR process. Representatives from the Division of the MoH were members of the National Maternal and Perinatal Death Surveillance (MPDSR) Committee and others were from the data management section of MoH.

### Ethics approval and consent to participate

The study was approved by the Makerere University Higher Degrees School of Medicine Research and Ethics Committee (SOMREC), # REC Ref 2018-001 and by the Uganda National Council of Science and Technology (UNCST), assigned number (UNCST, Ref SS4797). All study procedures were conducted as per the relevant guidelines and regulations. The study participant had to provide written consent before the interviews and they agreed to have audio recordings and dissemination of study results. The confidentiality of the participants was maintained by use of interviews numbers, no actual names within the transcripts and audio tapes would be destroyed as soon the data analysis is completed

### Study design and sampling

In order to explore the barriers and facilitators to MDSR, we conducted an explanatory qualitative study from March 2019 to December, 2019 to assess perceptions of the different stakeholders. 

Overall, 67 health workers and other stakeholders were purposively selected on the basis of their current or past involvement in conducting or planning MDSR, using maximum variation sampling to enrich the data. Of these, 24 participated in-depth-Interviews (IDIs), 15 in Key informant interviews (KIIs) and 28 in the 4- focus group discussion (FGDs). Table 1 shows the characteristics of the study participants. The stakeholders from Ministry of Health and other partners in Reproductive Health were people familiar with the MPDSR process.

**Table 1.** Characteristics of study participants who participated in KII, IDIs and FGDs.

Job Title	Age range	Mean Age	Range of years at work station	Gender		Previous MDSR training		Total
				Female	Male	Yes	No	
Specialist Doctors Obs-Gyn (8); One Anaesthesia, & two Pathologists (IDIs,)	30–53	47.0	6–26	3	8	7	4	11
Midwives (9 IDIs)	27–58	46.0	3 – 30	9	0	4	5	09
Resident Doctors (SHOs) (IDIs)	29–38	32.5	2.5–3.0	0	4	3	1	04
KII interviews for Leaders (Directors=3; Admin=2 & HOD=1)	42–58	50.5	5–30	4	2	3	3	06
MoH & RH-Partners (WHO, UNFPA, FHI-360, MANE, UMDPC, UMA) (KIIs)	33–60	49.4	3–20	2	7	8	1	09
4-FGDs:(SHOs=10; E/mid=6; R/Mid=7; Support team =5)	26–50	35.0	1–12	21	7	11	17	28
Total	**26–60**	**43.4**	**1–30**	**39**	**28**	**36**	**31**	**67**

 Most of the Internal stakeholders participated in IDIs. These were mainly health workers who were participating in activities of MPDSR committees. Additional participants were those available and identified by the Head Nurse or Clinical Head as having rich information on the maternal death review processes. Participants were contacted by phone call or physically by the first author who requested for an appointment on a day convenient for them to participate in interviews. The FGDs were conducted after the IDIs. The purpose of FGDs was to generate normative information about the MDSR process as well as health workers views and experiences regarding MDSR. We targeted people likely to be information-rich based on their experience after consulting the ward in-charges and people they work with. We felt that data from FGDs would enrich that from IDIs and KIIs to enhance data triangulation, since the MDSR processes requires team participation. Those who had participated in the KIIs or IDIs were excluded from the FGDs. In order to have homogenous groups, separate groups were arranged for specialists, residents, nurses/midwives and support staff (anesthetic officer, laboratory leader, pharmacy, stores, assistant -administrator). 

The external stakeholders who participated in the study were identified by NI working closely with the Ministry of Health (MoH) officers. All interviews were conducted in a private space to allow free communication and privacy.

### Data collection and informed consent process

The interview guides were pre-tested by the first author using one senior midwife, one obstetrician -gynecologist and one SHO. These initial interviews were used to refine data collection tools and data from this phase was excluded from the final analysis. All interviews and FGDs were conducted in English. The KIIs and IDIs lasted for about 30 to 50 minutes whereas the FGDs lasted 60 to 90 minutes. Each FGD had 6 to 10 participants.

Participants gave written informed consent to participate and to have interviews recorded. Open ended questions were used to explore their views on barriers and facilitators to timely MDSR at the Hospital. Other questions sought for top causes of maternal death at the Hospital and whether such deaths were preventable; participants’ opinions on protection of health worker from litigation and whether the information on review forms is protected from being used in Courts of Law in case of litigation. In addition, some questions to explore the domains in the theory of planned behavior (TPB) such as: attitude, towards performing MDSR, subjective norm (about usefulness and supervisor’s influence regarding MDSR); perceived behavioral control which would influence behavioral intention and finally behavior. The interviews were conducted by NI (an obstetrician-gynaecologist with public health training) and MM (a Social Scientist with public health training). Field notes were discussed to assess emerging issues. The study tool was developed using information available in the literature that guided the conceptual framework.

### Quality control

NI worked with another researcher (a social scientist) and note takers during the study to conduct interviews. The interview guides were pre-tested and refined to enhance clarity. All interviews were audio recorded and reference to field notes was also done during transcription. 

### Data analysis

The audio-recorded interviews (IDIs, KIIs, and FGDs) were transcribed verbatim by the note taker and prepared for analysis. Thematic analysis guided identification of emerging themes in an inductive manner. We followed the steps recommended by Braun and Clarke. i.e , “transcription; reading and familiarization; coding; searching for sub-themes; reviewing them; defining and naming themes; and finalizing the analysis”, ( Braun & Clarke, 2006; Damayanthi, 2019).

The initial coding (to identify meaningful phrases) and categories was done by NI and two research assistants with experience in qualitative research. These held de-brief meetings with the research team and discussed the codes to enhance trustworthiness of data. The coding team initially coded two transcripts together, then worked independently to identify upcoming themes and met regularly to get agreement and consensus on the codes generated. NI with the team read the transcripts a number of times in order to get familiar with the data. Transcripts were then coded manually using framework analysis in Microsoft-Excel (2010) computer program. The sub-themes were generated and aligned to TPB constructs of attitudes towards MDSR, subjective norms and perceived behavior control ( Ajzen, 1991) regarding implementing MDSR system Selected quotes from study participants have been used to present study findings..

## Results

This paper presents results from 67 participants; 24 IDIs from health workers at the Hospital, 15 key-informant interviews (KIIs) with external stakeholders and hospital managers; and 28 health workers who participated in 4 focus group discussions (FGDs). The 24 IDIs included 9 -midwives; 8-obstetricians-gynaecologists; 1-anesthesiologist, 2-pathologists and 4-residents (Senior House Officers).

Table 1 shows characteristics of the study participants. Their age range was 26 – 60 years with a mean age of 43.4 years. Most of the study participants had served for more than 5 years at their places of work, range in service of 1–30 years. Most of the participants were females 39 (58.2%). Many had not had exposure to MDSR training 31(46.2%) ( Table 1).

### Causes of maternal death as reported by the participants

All participants agreed that the burden of maternal deaths was high although most were not so sure of the exact number of women who die from the Hospital per month or per year. 

Most health workers mentioned post-partum hemorrhage, pre-eclampsia / eclampsia, sepsis and abortion complications as major causes of maternal deaths. However, some of the external stakeholders reported that delays of mothers at home, negligence of health workers and lack of emergency supplies were the major drivers. All study participants were in agreement that most of maternal deaths are preventable.

Importance of MDSR as perceived by the study participants: Almost all study participants scored the MDSR process to be very useful. Most of them proposed that all health workers should be brought on board to support the MDSR.

### Barriers and facilitators to maternal death surveillance and response

The major themes presented here as barriers and facilitators to implementation of MDSR were aligned to constructs of TPB as summarized in Table 2 (for barriers) and Table 3 (for facilitators) to MDSR respectively.

**Table 2.** Codes and sub-themes within the broader theme of barriers to maternal death surveillance and response (MDSR) aligned to Theory of Planned Behaviour (TPB).

Basic concepts/Codes	Categories (Sub-Themes)	Linkage with the Theory of Planned Behavior	Broader Theme
Inadequate knowledge	Inadequate knowledge/skills in MPDSR	Perceived behavioral Control	**BARRIERS**
Inadequate training in MDSR			
Lack of skills of MDSR			
Too many patients	Heavy workload		
Many deaths			
Inadequate number of health workers			
Inadequate institutional support	Leadership / Governance Challenges	Subjective norm	
Inadequate commitment of leaders			
Inadequate (Sub-optimal) leadership			
Limited interest by staff in MDSR			
False documentation	Fear of (Blame, litigation or criminalization)	Attitude towards MDSR	
Fear of blame (fear of arrest)			
Fear of litigation/ criminalization			
MDSR perceived as a policing game			
Low interest by staff	No response (MDSR cycle not completed)		
Failure to implement actions			
Implementing partners (IP) elsewhere			
Failure to follow up			

**Table 3.** Facilitators aligned to Theory of Planned Behavior (TPB) that strengthen implementation maternal death surveillance and response (MDSR) at National Referral Hospital.

Basic concepts/Codes	Categories (Sub-Themes)	Theory of planned behavior	Broader Theme
Orient HWs in concepts/ benefits of MDSR	Train / mentor all stakeholders on MDSR	**Perceived behavioral** **Control**	
Sensitize HWs on MDSR			**FACILITATORS**
Train all health workers on importance			
Train how to conduct			
Train administrators			
No blame game	Strengthen Leadership/ Governance and support blame free environment	**Subjective norms**	
Not punitive			
Collective responsibility			
Committed leadership			
Address leadership issues			
Change attitude			
Provide refreshments during meetings	Create Incentives for meetings	**Enhance attitudes to MDSR**	
Identify motivators			
Give an allowance			
Reward people			
Keep meetings short			
Regular committees	Strengthen / Create more committees		
Strengthen teams			
Orient committee members			
Conduct weekly meetings			
Orient all new members e.g SHOs as soon as they come			
Feedback to providers	Complete the MDSR cycle		
Complete the cycle			
Implement actions			

Under the theme of “barriers to implementation of MDSR” at the Hospital, the study findings revealed barriers in all the three constructs of the TPB as explained in the following sub-sections.

### Perceived lack of behavioral control

Under this domain, study participants identified inadequate knowledge and skills plus heavy work load as the major barriers affecting the implementation of MDSR.

***Inadequate knowledge and skills about MDSR*.** Study participants cited knowledge gaps and inadequate skills as one of the barriers to implementation of MDSR. Participants expressed difficulties in filling MDSR forms and the death notification process especially health workers that had not been trained as FGD participants explained;

“… *it feels [seems] that most do not know when to do the notification* (FGD-3- Enrolled midwives)..“

*….Most of us health workers have not been enlightened about this…there is knowledge gap because….no training of health workers (FGD-4-Registered midwives)*

When someone lacks the knowledge and skills, it becomes hard to take charge of something or behavior change. Some participants reported that at one stage they did not know what to do until they were taught about MDSR processes as one FGD participant explained:

“… *the other thing that actually kills us health workers is knowledge gap. I believe a person tries to fill one of those audit forms the way they used to call them I used to fear actually to fill it, until I was taken through it and I got to know it is something easy that I can actually lead a team… and …. but before that you don’t fill them because you have no idea of how to put things right (FGD-1-SHOs)*

***Heavy workload as a barrier to MDSR implementation*.** Most study participants mentioned heavy workload as a major barrier to MDSR. Most participants echoed the high burden of patient numbers in relation to the limited number of health care providers. Some participants reported that the current staffing level is about one third of the expected (staff capacity of 320 / 900). Others noted lack of time to do the maternal death reviews because of competing schedules, which led to postponing the reviews, often creating many unreviewed or delayed reviews of deaths. This greatly interferes with control domain. They felt that it is beyond them as reflected in some of the quotes below.

*“…overwhelming number of patients… there might be this other team who [report] the house is very bad…They are supposed to audit and really they cannot leave other mothers again to die and then begin auditing so they put it aside, at the end of the day they go in exhausted so… they keep on postponing and when somebody take long (without doing the reviews), they tend to forget certain things” (IDI-Resp-02, Midwife)*

Many health workers expressed feeling work overload which often emerged as an area of conflicting interests to either leave work at the unit or participate in MDSR meetings. This setting characterized by inadequate number of health workers, who are overworked and with knowledge and skill gaps hindered implementation of the MDSR.

### Unfavorable subjective norms as a barrier to implementation of MDSR

Regarding unfavourable subjective norms, findings revealed leadership and governance challenges such as inadequate institutional support for MDSR, inadequate commitment by leaders and low interest by staff affecting implementation of MDSR. 

***Inadequate leadership and governance*.** Some study participants cited that some leaders appear unconcerned, indifferent or ambivalent regarding implementation of MDSR which discouraged health workers. Participants reported that leadership at various levels is very important (i.e., Right from the politicians, the Ministry of Health, the Directors, Administrators, In-charges of the wards, and head of Department teaching side are all critical) for effective implementation of MDSR. Participants believed that once the leadership supports, provides the resources and embraces the entire process, then high levels of MDSR will be performed. This would enhance better survival of patients. When leaders have interest, health workers would be encouraged and motivated to stop doing MDSR as a formality but instead as a routine practice as exemplified by respondents:

*“…. maternal death review …[looks] as if it is just a formality whether you do it or you don’t do it you are not going to see anything different (Resp-9 -SHO-IDI)*”

“ *……leadership not showing interest is a problem… …. because you know these facilities are very busy and if leaders do not structure a way of having the deaths reviewed on a regular basis so that there is no backlog…(Resp-41-MoH, KII).*

“… *I think first and fore most there should be buy-in from leaders and key stakeholders... to walk the MDSR talk…… (Resp-14-Leader - KII)*

Another governance challenge that was often expressed by study participants as a barrier to the implementation of MDSR was erratic supplies [shortage of supplies] which tend to demotivate the service providers. Recurrent shortage of supplies such as sutures, magnesium sulphate, blood products and so on was understood by some health workers to imply that MDSR was not important.

*"..the key barriers is relative supply like I will overly emphasize emergence preparedness and complication readiness of the facility in terms of you know yes the supplies, sundries, drugs…" (Resp-21-OBS-GYN-IDI)*

 Other health workers mentioned lack of incentives such as allowances and refreshments during MDSR meetings as a barrier.

“… *lack of incentives, the incentives basically, those because we come expecting and they readily demanded but we are doing all this work, we have sat here for two hours….but nothing* ” (Resp-012- IDI)

In addition, some participants cited insufficiency of the hard copies of death notification and audit forms and failure to have an electronic system to submit the required information to MoH as a hindrance to implementation of MDSR.

### Unfavorable attitude of health workers and managers towards MDSR

The main sub-themes reflecting unfavorable attitude towards MDSR in study participants narratives were 1) fear of blame and 2) incomplete MDSR cycle.

***Fear of blame as a barrier to MDSR implementation*.** Fear of blame was reported as a major barrier to MDSR process especially by senior house officers (SHOs). They felt that leaders and other health workers insinuate that they are the cause of deaths. Others reported that writing names on maternal death notification exposes them to blame. Furthermore, participants quoted stories of health workers who were arrested after a maternal death. The fear is even more in the event of a maternal death of a politician or someone related to influential people. Fear of blame is not only with the team in training, but also with some of the senior health workers and leaders. Some people get worried that if they notified a death within the first 24 hours, probably it would backfire on them if there were enquiries from the higher level, as exemplified by quotes from respondents:

*“Yeah, if you think squarely, you will be blamed for this maternal death you wouldn’t be motivated in participating … No, that word who caused the death, actually …. last night -ok you killed a mother last night. Somebody asking you the question, you killed one last night, there is a way it just makes you feel, eh, (pause) it means I was the cause of death of this one (FGD1-SHOs)*

*“…. people are not free, I think that’s fear. Fear is something we see very often because it’s like it’s supposed to be a process that is blame free, but in the end, it almost appeared like it’s a blame game”. (Resp-9-SHO, IDI).*

*“…there is fear even reviewing the maternal deaths because in most cases you will identify there is a gap, [in care] maybe if we have acted, so this we could have saved this mother from what, (pause) from dying, so that fear alone also is a deterrent especially you know……but if the law asked us to produce the maternal death review form [from] the health workers, [we fear]…..we are not protected” (Resp-4, Obs-Gyn, IDI)*

One of the obstetrician/gynecologists reported that fear is still real, relating it to a letter from a past Minister of Health which instructed the police to investigate every maternal death.

“…. *I remember when the president wrote a letter instructing the ministry, copy to the police, copy to so many people, (to these RDCs) instructing that every maternal death should be investigated by police and some of our colleagues were actually arrested when a maternal death happened because they had the authority…… because of that fear most people fear to report maternal deaths ( Resp-4, Obs-Gyn, IDI,*

*“…sometimes we try to point fingers and even blame colleagues ..(respondent laughs)..(Resp-1-Midwife-IDI).*

Some study participants felt that influential people abuse the MDSR process and induce fear among healthcare providers about notifying deaths, as it might involve court proceedings:

*“Some patients might be highly influential/political or those who have too many attendants. and most staff feel, they will be held responsible [for] that death* (FGD-4-Registered Midwives)

*“ …what most people have been fearing is that supposing I notify, the ministry of health is going to ask the head of the institution, there is a death which has happened in your place ok and some of those messages coming back from the ministry of health are not supportive ….. the head of the institution says but who told you to notify?... you know, because it [may have] has the legal implications (Resp 6, Obs-Gyn-IDI)*

*“…… the forms [filled death review forms] might be used in court against them and... it’s hard to remove those fears especially due to deaths caused [gaps in care] as a result of many patients”. [FGD3-Enrolled midwives]*

However, few participants reported that MDSR is not a punitive process for the individual, but can be used to correct errors when things can go wrong within the system, and suggested that in such a situation, call a colleague and correct him or her, as reflected in the quote below.

“ *….MDSR process is not a blame system but you can just pick and talk to an individual of whatever happened and maybe you can also review the system …..see whether it is the system which failed …., to manage the patient for example like theater you can have a system where there very many patients and you cannot put in an individual and sometimes happens before she goes to theater, sometimes it is blood, sometimes it is human resource so it [MDSR] gives you time to identify what went wrong so that it is corrected..” (Resp-2- midwife, IDI*)

### Incomplete MDSR cycle as a barrier

Implementation of recommendations is partly governed by attitude at various levels although availability of resources may also affect it. Most of the participants reported that failure to implement recommendations is a major barrier to MDSR. Completion of the cycle is considered to be a critical step, but support for this even from Ministry of Health was perceived to be low, as reflected in the quotes below

*“I want to start with the biggest barrier being lack of implementing the recommendations (FGD1, SHOs).*

*“Failure to implement recommendations, minimal level of commitment by the staff members, work load…” I think also other barrier beyond the health facility we are not getting adequate support from the ministry”. (Resp 4, Obs-GYN, KII)*

***Negative attitude as a barrier to MDSR under personal attitude was also reported*.** Some of the participants cited negative attitude of health workers as a critical barrier to MDSR. Some felt that commitment is not from all members partly because of negative attitude, despite all the other challenges. However, some of the participants noted that health workers do not take MDSR processes seriously due to heavy work load, inadequate knowledge and skills and others perceiving the process as punitive.

“ *The ones that I’ve sat with are seeing it good but the people who are not involved in sitting in those meetings for reviews, they still have a negative attitude because whenever you call them come let’s go for review, aa…aah, they still think that it is a punitive review” (IDI, Resp 1, Midwife).*

*Negative attitude of the health workers towards the review hinders the process. (Resp- 6, Obs-GYN, KII) “*.

“Some *of my peers have…, they still have a negative attitude. Yes, so the training is all in all for all because it is very important for all of us to have the knowledge and also to participate in the reviews and use it for continuous improvement”* (Resp 28, Obs-Gyn, *IDI).*

Lack of adequate champions to spearhead implementation of MDSR and differences in exposure and understanding of the process are other key barriers mentioned.

*“..there are, a few people who have dedicated themselves to making these reviews and everybody knows that it is very important, it is a recommendation by ministry of health but people have to dedicate themselves to make sure that these reviews are done timely*.’ ( *IDI, Resp 1, Midwife).*

*"I think one of the challenges could be that maybe not everybody is well informed about it or not everybody in the units has probably received it with the same kind of importance…” (Resp-25, Obs-Gyn-IDI)*

### Facilitators to strengthen MDSR at the National Referral Hospital

Under the theme of “facilitators to implementation of MDSR” at the hospital, again the facilitators were aligned to the three main constructs of the TPB as explained in the following sub-sections.

***Training and mentorship to enhance perceived behavioral control as a facilitator of MDSR*.** Training in MDSR being essential in harmonizing the review process was echoed by most participants. In addition, most participants voiced the need to bring all stakeholders onboard through training. This would enhance behavioral control through enhanced skills and knowledge most likely performance of MDSR. One of the key facilitators was that some of the people who were already trained, although few, were conducting some reviews the facility. Training would enhance understanding of objectives of MDSR, the guidelines or policy that explains their scope of work, terms of reference, benefits of conducting the reviews, concepts and use of MDSR data to improve health systems for maternal health care. Important to note is that including administrators in the training was reported to be critical to enable them address the health systemic issues that are identified to improve outcomes.

*“..the important thing would be to train people, train people on how to conduct [MDSR], train people on how to utilize results of the review process, yes to me I think that would be the most important”.* (Resp-028- Leader- KII)

*“We need to get our hospital administrators trained with the health service providers and to take lead…. rectifying things which are supposed to be done; for example, like when somebody dies of a situation without blood [Blood not available]], we must look at why and how to get that blood so that it doesn’t happen again”.* (Resp-002- midwife-IDI).

Training comes with other things and not just how to conduct MDSR. It comes with information on why it is important, training in soft skills, communication skills, leadership, and how you should handle issues where the MDSR cycle is not completed. It is the shared experiences that come with the training that cause trainees to have a change of mentality about MDSR. Some participants reported that including MDSR training in teaching institutions as a pre-service course unit would be a good strategy to get the students get MDSR embedded in their maternal health knowledge and skills acquisition. In addition, participants recommended that legislators also need training regarding the importance and principles of MDSR.

***Favorable subjective norms as a facilitator to MDSR implementation*.** The main sub-themes under this were strengthening governance /leadership and supporting a blame free environment

***Strengthening governance /leadership as a facilitator to MDSR*.** Most study participants identified strengthening leadership/governance as key to MDSR. If the leaders are committed to it, they will encourage people to conduct the reviews. In addition, most of our participants mentioned that acting on the recommendations to address gaps identified depends on leaders or administration of the hospital. Good leadership at various levels, also acts as cheer leaders when the leaders are committed to seeing the MDSR process working, functioning and producing results. The leaders should be empowered, supported to be in control and to support hold each other accountable at various levels of the health system for a holistic system strengthening.

*“…this is a block [administration block] that articulates all the others, it does the decision making, it decides what happens [and] when. Now when you have decided at that level, it is very easy to tackle an issue because if you have a good leadership and governance, you will somehow have good supplies, … good health systems, you know they will decide when people train and when they don’t, they will decide which health workers they have so I think leadership and governance is the first block we need to tackle. It will help us articulate all the others to have a good death surveillance system”.* (FGD-4_Senior Midwives).

*“…governance is very critical, if people supply and they don’t follow up to see what they’ve supplied, if health workers absent themselves and no one follows up to find out why…. The functionality of the hospitals depend on governance and governance is a structure right from up to your immediate boss.* (Resp-6, Obs-Gyn -KII)

Furthermore, top leadership should ensure that the seniors and all people on the teams managing patients take charge. Currently the process is being driven mainly by junior doctors who would not impact on the decision-making and demand for actions on the recommendations made.

“… *the real top leadership ..[get involved] or probably where I am should ensure that the leaders of the teams managing patients take charge… because what is happening currently this [MDSR] process is being driven mainly by senior house officers, they really first of all are students” (Resp-14-Facility leader, KII)*

***Creating a blame free environment as a facilitator to MDSR and enhancing accountability*.** Overall, the participants were of the opinion that there were few facilitators to MDSR at the time of the interviews in 2019/2020. However, one of the leaders mentioned that some MDSR related meetings were taking place and reportedly non-punitive since people were free to express themselves, without fear of being blamed.

*“…they are very objective meetings, people are made to feel free to express themselves, to talk about how they feel, to talk about what happened and because they come with that attitude of am not going to be blamed, they even accept the mistakes where they happened (Resp-023-Facility Leader-KII)*

However, most participants were of the opinion that blame was rampant and it should be eliminated while at the same time ensuring accountability and responsibility of one’s actions. MDSR committee members should be reminded of the importance of separating blame from the actionable recommendations. Participants considered that there are actions to address absconding from duty causing a maternal death as much as there are actions to address a lack of clinical skills; albeit different. While the recommended action would be to re-train or give skills to the latter, for the former, the recommendation could include a harsher action such as expulsion from the institution. But in all this, the action is explained to the provider and is not blamed but rather s/he is taking responsibility for her actions.

*“.. but what I can say is much as we said it is not a blame game we don’t want to promote negligence at whatever side be it administrative, be it on the clinical side there are cases where we detect negligence on the side of the health care providers if the law comes in, let it take its course as long as thorough investigations are made to prove beyond reasonable doubt that someone is guilty of negligence so the audits should not be a reason for people to commit careless mistakes moreover with the lives of others because of no blame” (Resp-23-Facility-LeaderKII)*

*“..not covering up on those in the wrong is important … that’s why one of the weaknesses for the maternal audits, and which we have been quarrelling with, this business of saying that, let us not blame anybody. Let us not blame the wrongdoer…, let us correct what didn’t go right. …. if it is a system failure, let the medical superintendent or the CAO who didn’t provide the money be taken on. We cannot just sit on maternal death like that” (Resp-029- External Stakeholder-Leader -KII)*

### Favourable attitude of health workers as facilitator to MDSR

The main sub-themes reflecting favourable attitude towards MDSR in this study were: (1) provide incentives/motivation for meetings (2) completion of the MDSR cycle (3) strengthen or create more MDSR committees as reflected in Table 3.

***Provide incentives/motivation*.** Some participants reported a need to provide incentives to conduct MDSR which may not necessarily be monetary. Incentives could include refreshments during the meetings and an office designated for the purpose. Participants echoed that understanding the importance of the death review is in itself a motivation. Because care providers get to know why mothers are dying and are then able to make recommendations to address the gaps to prevent similar deaths in the future. In addition, some participants reported that refreshments during the meeting will enhance the quality of the discussions.

*“…we should be having the money to make sure that those critical life-saving things must be there. ..what is the opportunity cost for me coming to sit in your MDSR meeting of which I know all meetings we have sat in, no response has been done there is no effect, no impact* (Resp-6_Obs-Gyn-IDI).
“… *we could sit comfortably because you are not hungry, you concentrate, so they should give them some refreshments, simple ones*” (Resp-018_IDI)

***Completion of the MDSR-cycle as a facilitator*.** Completion of the cycle is considered as a critical step. Failure to implement recommendations was identified as a major barrier. Thus, all efforts should be geared to prevention of death from similar circumstances through implementation of the appropriate recommendations. Improving quality of care to prevent similar deaths (mothers and newborns) is the major goal of death reviews. Most participants believed that response at various levels is urgently needed. Some reported that, one of the major disincentives was repeated meetings without realizing any impact.

*“…we need to see the implementation of the recommendations as a big motivator because, personally me I look at it as wasting my time, just like the morning meetings am sorry to bring it up. The same song is done from Monday to Friday, we sing. ..but nothing changes. So you actually feel you are wasting your time (Resp-21, Obs-Gyn-IDI)*

*“We should implement ..for example ..we need a medical doctor either or an intern to be placed at least in the zero post-operative….[.it is] critical because those who going to get stable [post c/section] their survival is in our hands because [close] monitoring is needed .. (FGD-Senior House Officers)*

*“…I think we should be able to freely. discuss it and constructively look for the response .,. what could we [do] at our level, individual capacities…such that next woman who walks through the cycle [system] does not have to suffer this same fate of death” (Resp-013-KII-Leader)*

***Strengthen or create MDSR – Committees as a facilitator*.** Functional committees should have committed volunteers; members who feel that they can make a difference or they believe can generate information or data to make a change in the health care system. The committee composition should have different cadres and members should be able to seat regularly and have champions to spear head and enthuse the reviews.

*“…we need to orient them [ training committee members], we need to work on the aspect of when do they seat like what has been directed in the guideline and policy and then all that is explained in their scope of their terms of reference”. (Resp-012, Obs-Gyn-_IDI)*

Some mentioned that it would not be very difficult to meet when people are committed to it and health workers are also encouraged to do their best. Therefore, what is most important is to have an active MDSR committee, and preferably, this committee should set a specific day for review. The meeting should preferably take place in the department where the death occurred instead of meeting in the administration block. This avoids people failing to attend because they deem themselves busy.

“…. *I think that maybe we as health workers, we also need to play our cards rightly. Be on duty at the right time and when you are supposed to be on duty do the right thing.* (FGD-2- support team).

*“…There should be a particular day, and a must day that the committee should sit every week. … say Friday because sometimes the death maybe on Thursday. So, it’s an advantage for Friday you are closing the week; and then, the other workload through the week would have been summarized.* (FGD4-Senior midwives)

## Discussion

Our study findings revealed a favorable perception of the MDSR process as either very useful or useful. However, they mentioned a number of barriers that hindered successful implementation of MDSR at the National Referral Hospital. These were aligned to the major constructs within the Theory of Planned Behaviour (TPB). Regarding perceived control, most participants reported inadequate knowledge /skills and heavy work load as major barriers to MDSR implementation. Inadequate leadership/ governance a key barrier reported under unfavorable subjective norms. Fear of blame and failure to complete the MDSR cycle were the main barriers under the construct of attitude. Inadequate skills to conduct MDSR, work load, fear of blame and litigation and failure to implement recommendations have been reported in some studies as hinderances to maternal and perinatal death surveillance and response practices in Uganda ( Agaro *et al.*, 2016); Rwanda ( Tayebwa *et al.*, 2020), Tanzania ( Kinney *et al.*, 2020).

Relatedly, the multi-stakeholder group of participants reported key facilitators to MDSR aligning them to the TPB. Regarding perceived behavioral control, our findings revealed a need to train all stakeholders. The participants voiced that all stakeholders should be on board so that they acquire the skills, importance of MDSR and receive terms of reference. The facilitators under subjective norms are strengthening leadership and governance which moves along with efforts to eliminate blame. These are the cornerstones to enhance oversight of MDSR, accountability and implementation of recommendations at the various levels. Then under favourable attitude construct, the participants reported creation of incentives for MDSR meetings, strengthening or foaming more functional committees and completion of the MDSR cycle as key facilitators. All these require commitment and political will to support funding and streamline the legal framework to counteract the fear of blame. These findings are in agreement with studies in Ethiopia where strong political will, efforts to streamline the legal frame work and strengthening leadership/ governance enhanced MDSR uptake ( Abebe *et al.*, 2017). Training and mentorship to build the capacity of service providers are greatly encouraged as stipulated in the WHO guidance ( World Health Organization, 2021). A secondary analysis study on lessons from 10-country case studies (both low, middle and high -income countries) on MDSR, Smith and colleagues showed that the major drivers for successful implementation were adequate legal framework, no shame, no blame culture, government and political commitment ( Smith *et al.*, 2017b) which further support the facilitators reported in our study.

The barriers and facilitators were aligned to the domains within the Theory of Planned behavior (TPB), ( Ajzen, 1991) which emphasizes that desires inform motivations, which then inform intentions and eventual behavior. In this study, participants’ desire to see maternal mortality reduction was a critical motivator to participate in MDSR activities. However, issues of inadequate skills, fear of blame and litigation could partly explain the sub-optimal participation. The lack of perceived control due to inadequate knowledge, inadequate resources to perform timely MDSR, workload and challenges with technology would greatly affect the feeling related to control and actual performance. However, with enhanced training and mentorship, plus strong leadership and governance to ensure provision of incentives, availability of tools and appropriate technology would enhance perceived control. When we critically analyze the domain of subjective norms, aspects of peer influence, from either senior or junior colleagues, side are also likely to be addressed by supportive leadership and governance. Other researchers have explored the utility of TPB in a metanalysis to predict behaviour in health-related research such as predicting alcohol consumption and nurses taking care of patients who are involved in binge-drinking ( Cooke *et al.*, 2016; McEachan *et al.*, 2011). They recommended that the constructs of attitude and perceived control in TBP are still useful in predicting intention to perform a particular behavior, ( Cooke *et al.*, 2016; McEachan *et al.*, 2011). Ajzen defends the TPB further by stating that it requires to understand the TPB and related constructs : attitude, subjective norms, perceived behavioral control, intention and behavior itself but not just looking at its graphic presentation alone ( Ajzen, 2015).

The WHO guidelines and related support mechanisms have been developed and made available to Ministries of Health and online, ( World Health Organization, 2021). These regional technical guidelines aim to standardize and improve national MPDSR processes. However, more studies to asses impact of implementation of maternal and perinatal death surveillance in general are needed. This also may be augmented by national, district, regional and health facility committees, as well as by a technical working group to advise on how MDSR is planned, implemented and evaluated.

Our findings revealed that all core elements/ steps of MDSR cycle are important. However, for improvements to occur, participants believed that implementation of recommendations (Response), eliminating blame, training to enhance skills and knowledge, plus strong leadership are all critical. All these aspects should be emphasized during training of all healthcare providers, and stakeholders. It important to remind all stakeholders that all steps of MDSR (timely notification and review of deaths; correct identification of gaps, developing feasible recommendations, implementation as well as monitoring and evaluation) are all important if the process is to achieve the intended goals ( World Health Organization, 2021).

Our findings revealed the critical role of governance and leadership for successful implementation of MDSR. Often described as the most influential factor in shaping organizational culture, effective leadership is critical at all steps of the MDSR process ( Mathole *et al.*, 2018). Supportive leadership enhances the software aspects which may be applicable to MDSR too. Though engaged leaders are widely recognized as an enabler, necessary leadership traits from individuals and critical thinking or problem-solving skills are also crucial ( Mathole *et al.*, 2018). Thus, more needs to be understood on what motivates these leaders, what skills are needed, and how to nurture champions. In addition, there is need to innovatively equip them with leadership skills to discourage blame and other negative influences to enhance MDSR. Nonetheless, there is need to identify how MDSR can enhance leadership in health, responsibility and accountability among all stakeholders at various levels ( Gilson, 2016); ( Mathole *et al.*, 2018); ( Schneider *et al.*, 2020). The complex interplay of networks between health system levels, different sites and different role players influences MDSR implementation ( Lewis, 2014; Raven *et al.* 2011).

This study revealed failure to complete the MDSR cycle, characterized by not implementing actions from death reviews as a key barrier. In line with our findings, other studies have noted that the MDSR cycle must be completed by implementing actions in order to trigger iterative cycles of improvement as a culture of success to improve outcomes ( Bandali *et al.*, 2016; Kinney *et al.*, 2020; Lewis, 2014; Moodley *et al.*, 2014; Pattinson *et al.*, 2005). Notwithstanding, is the importance of a holistic approach to weave in the various health system building blocks recommended by World Health Organization (WHO) to improve quality of health care to reduce deaths. Successful implementation of MDSR should embrace integration within the health system building blocks framework (service delivery, health workforce, health information systems, access to essential medicines and equipment, financing, and strong leadership/governance) ( Manyazewal, 2017). All these require a motivated team to prioritize what should be tackled first depending on available resources.

### Strengths of the study

Data was obtained from multiple sources using IDIs, KIIs and FGDs and involving internal and external stakeholders. This diversity of study participants ensured complementarity of ideas that enriched the content and facilitated triangulation. In addition, the use of TPB helped to identify barriers and facilitators to MDSR implementation across the domains of the framework. The study site is a National Referral and Teaching hospital whose staff are likely to have a lot of influence elsewhere within the country. Strategies to strengthen this quality improvement process of MDSR in this setting is likely to have influence in other parts.

### Limitations of the study

Most of the study participants were from one high volume setting. Thus, the results may not be generalized to other settings. However, the health system issues encountered in the National Referral hospital may not be different from other hospitals since more than 50% of the patients at the national referral are referred. In fact, issues such as lack of supplies, skills and fear of blame could be worse in lower health units.

## Conclusions and recommendations

The study sheds light on barriers and facilitators to implementation of MDSR, which if addressed would enable stakeholders to fully embrace MDSR. This could focus on how MDSR is implemented, particularly looking at mitigating the barriers and enhancing facilitators to reduce maternal mortality.

Efforts to enhance knowledge and skills of various health workers in MDSR processes; eliminating blame, implementing recommendations and protection of health workers and data from audits from being used in courts of law in case of litigation as well as strengthening leadership are critical for a successful MDSR process. Successful implementation of MDSR requires use of a health system wide approach for impact.

## Data Availability

Information related to maternal death surveillance is deemed sensitive due to a lot of fear of blame. However, de-identified information can be availed from the corresponding author on reasonable request.
