# Supplementary material for: Barriers and facilitators to maternal death surveillance and response at a busy urban National Referral Hospital in Uganda
Source: Open Res Afr. 2023 May 30;5:31. Originally published 2022 Sep 14. [Version 2] doi: 10.12688/openresafrica.13438.2 (PMC10280031; doi:10.12688/openresafrica.13438.2)
Supplement: Peer review history

# Peer review: Reviewer response for version 2

**DOI:** 10.21956/openresafrica.15161.r29749

**Published:** 2023-06-13

**Authors:** Wendy J. Graham

**Affiliations:** 1 Department of Infectious Disease Epidemiology, London School of Hygiene and Tropical Medicine, London, UK

The authors have comprehensively addressed my comments and I am completely satisfied with their responses and the amendments made to their paper.

Is the work clearly and accurately presented and does it cite the current literature?

Yes

If applicable, is the statistical analysis and its interpretation appropriate?

Yes

Are all the source data underlying the results available to ensure full reproducibility?

Yes

Is the study design appropriate and is the work technically sound?

Partly

Are the conclusions drawn adequately supported by the results?

Partly

Are sufficient details of methods and analysis provided to allow replication by others?

Yes

Reviewer Expertise:

I am an obstetric epidemiologist, with 30+ years of experience in the measurement of maternal mortality, including the use of MDSR, and have worked in partnership with colleagues in many low and middle-income countries.

I confirm that I have read this submission and believe that I have an appropriate level of expertise to confirm that it is of an acceptable scientific standard.

# Peer review: Reviewer response for version 2

**DOI:** 10.21956/openresafrica.15161.r29750

**Published:** 2023-06-05

**Authors:** Joseph Ngonzi

**Affiliations:** 1 Department of Obstetrics and Gynecology, Faculty of Medicine, Mbarara University of Science and Technology, Mbarara, Uganda

I have carefully read through the responses to the comments I raised. The author has adequately addressed the concerns raised and I am satisfied that the comments have been attended to with great detail. The manuscript reads much better in the current form.

 I do not have any further comments or questions concerning the revised manuscript.

Is the work clearly and accurately presented and does it cite the current literature?

Yes

If applicable, is the statistical analysis and its interpretation appropriate?

Yes

Are all the source data underlying the results available to ensure full reproducibility?

Yes

Is the study design appropriate and is the work technically sound?

Yes

Are the conclusions drawn adequately supported by the results?

Yes

Are sufficient details of methods and analysis provided to allow replication by others?

Partly

Reviewer Expertise:

1) Postpartum sepsis 2) Maternal mortality 3) Placental clinical and pathology 4) High risk obstetrics 5) Artificial intelligence in cervical cancer pathology

I confirm that I have read this submission and believe that I have an appropriate level of expertise to confirm that it is of an acceptable scientific standard.

# Peer review: Reviewer response for version 1

**DOI:** 10.21956/openresafrica.14587.r29317

**Published:** 2022-11-21

**Authors:** Joseph Ngonzi

**Affiliations:** 1 Department of Obstetrics and Gynecology, Faculty of Medicine, Mbarara University of Science and Technology, Mbarara, Uganda

The abstract reads well. It is well summarized and the most important components of the research are well outlined. It would be great to include a sentence on barriers to maternal death surveillance and response. It is good to read some recommendations in the abstract section under the conclusions.

 It would be a good idea to write a sentence on barriers and facilitators to maternal death surveillance and response as known from the literature, in the introduction. Please state the national MMR for Uganda at the time of the research.

 You made mention of MDGs in the background, it would great to align your background with SDG 3 too. I recommend that the introduction is concluded by stating the aims of the research study.

 Under the methods section, it would great to have a glimpse of the maternity load at the facility where the research was done. What is the MMR at the national referral facility? What is the annual total delivery numbers at the facility? You mention that the external stake holders were people familiar with the MDSR process. Please give examples of those external stakeholders who were involved.

 Why is table 1 on Characteristics of study participants who participated in KII, IDIs and FGDs in the methods section rather than the results section? I thought it’s part of the results.

 Table 2 and 3 present the codes and subthemes of the barriers and facilitators in a well aligned manner to the theory of planned change. In the text write up that follows the tables, please make the subsections uniform. Some of them are bolded while others are not.

 The discussion is fairly well summarized. There’s some attempt to elucidate the differences and similarities between their study and other quoted studies. Under the study strengths, you state that "the study site is a National Referral and Teaching hospital whose staff are likely to have a lot of influence elsewhere within the country". Is it the national referral status that makes the impact influence or the stakeholders you interviewed, including policy makers who have more influence on the rest of the facilities in the country?

Is the work clearly and accurately presented and does it cite the current literature?

Yes

If applicable, is the statistical analysis and its interpretation appropriate?

Yes

Are all the source data underlying the results available to ensure full reproducibility?

Yes

Is the study design appropriate and is the work technically sound?

Yes

Are the conclusions drawn adequately supported by the results?

Yes

Are sufficient details of methods and analysis provided to allow replication by others?

Partly

Reviewer Expertise:

1) Postpartum sepsis 2) Maternal mortality 3) Placental clinical and pathology 4) High risk obstetrics 5) Artificial intelligence in cervical cancer pathology

I confirm that I have read this submission and believe that I have an appropriate level of expertise to confirm that it is of an acceptable scientific standard, however I have significant reservations, as outlined above.

# Response

**Published:** 2023-03-17

**Authors:** Imelda Namagembe

**Affiliations:** Obstetrics and Gynaecology, Makerere University, College of Health Sciences / Mulago National Referral Hospital, Kampala, Uganda, Uganda

**Response to reviewer report from Joseph Ngonzi ** Department of Obstetrics and Gynecology, Faculty of Medicine, Mbarara University of Science and Technology, Mbarara, Uganda Thank you so much Prof Joseph Ngonzi for having created to review the manuscript. The response to the concerns raised is as follows.

1. The abstract reads well. It is well summarized and the most important components of the research are well outlined. It would be great to include a sentence on barriers to maternal death surveillance and response.

 Thank you for this kind note. We have inserted a sentence on possible barriers “Implementation of MDSR is still suboptimal in LMICs due to barriers such as inadequate knowledge, skills and leadership to support MDSR”. Please Professor Ngonzi, there is a word limit in the abstract version, thus it difficult to add more.

1. It is good to read some recommendations in the abstract section under the conclusions.

Thank you so much for the comment We have discussed this aspect with some of the co-authors and agreed that the sentence in the abstract starting with “Efforts to strengthen…” is a recommendation, thus we added the word “recommendations” as shown below. Recommendations: Efforts to strengthen MDSR for impact should use health system responsiveness approach to address the barriers identified, constructive participation of health workers to harness the facilitators and addressing the required legal framework”

1. It would be a good idea to write a sentence on barriers and facilitators to maternal death surveillance and response as known from the literature, in the introduction.

We have inserted in the introduction paragraph 2. Some studies have reported barriers such as inadequate training, lack of financial motivation and high turnover of staff (Agaro et al., 2016, Smith et al., 2017, Abebe B et al., 2017). However, training stakeholders involved, a strong political will and supportive legal frame work that minimizes fear of litigation are some of the reported facilitators for MDSR (Abebe B et al., 2017).

1. Please state the national MMR for Uganda at the time of the research. You made mention of MDGs in the background, it would great to align your background with SDG 3 too.

Thank you for the comment. We have edited the sentence that has MDGs and added a clause to align with SDG-3 in that same paragraph No.1 of the introduction as stated here. Uganda had a modest reduction in its maternal mortality ratio (MMR) over that millennium development goals (MDG) period, i.e. from 506 per 100,000 live births to 336 per 100,000 livebirths (Uganda Bureau of Statistics and ICF, 2016) was the official MMR during the study period too. This MMR of 336 per 100,000 livebirths is still way below the national sustainable development goal (SDG) target of ≤140 to contribute to a global one of ≤ 70 per 100,000 livebirths by 2030 (Nations, 2015, McArthur et al., 2018).

1. I recommend that the introduction is concluded by stating the aims of the research study.

Thank you Prof for the comment. This statement has been inserted at the end of the introduction: “Therefore, in this study we purposed to explore the barriers and facilitators to MDSR implementation and also obtained information on what the participants proposed as priority interventions to strengthen the process”.

1. Under the methods section, it would great to have a glimpse of the maternity load at the facility where the research was done. What is the annual total delivery numbers at the facility? What is the MMR at the national referral facility?

Thank you for the comment. The concern has been addressed in the method section and also inserted here. The study setting is one of the sub-Saharan Africa’s busiest maternity Centre (Nakimuli et al, 2016). The total number of annual deliveries ranged from 23,000 – 27,000 over the three years 2016 to 2018 (Namagembe et al, 2022). The institutional MMR ranged from 500 to 600 per 100,000 livebirths.  In addition, this Centre contributes to the biggest number of deliveries plus maternal and perinatal deaths in Kampala District but performance of maternal death reviews (audits) was still low during the studied period (Namagembe et al, 2022).  

1. You mention that most of the external stakeholders were people familiar with the MDSR process. Please give examples of those external stakeholders who were involved.

Thank you for this. Examples of the external stakeholders were representatives from Ministry of Health Reproductive Health division, World Health Organization, UNFPA, Kampala Slum Maternal and Newborn project as was stated on page 3 of the manuscript in the sub-section that described study participants.

1. Why is table 1 on Characteristics of study participants who participated in KII, IDIs and FGDs in the methods section rather than the results section? I thought it’s part of the results.

Thank you for the query. During writing and initial submission of the manuscript for publication, Table 1 on characteristics of study participants was inserted in the results sections, however, during type-setting or formatting of the manuscript by the journal’s team, it was moved to method section. Some journals do it.

1. Table 2 and 3 present the codes and subthemes of the barriers and facilitators in a well aligned manner to the theory of planned change. In the text write up that follows the tables, please make the subsections uniform. Some of them are bolded while others are not.

Thank you for raising the concern. It has been addressed and bolding made uniform in the relevant subsections.

1. The discussion is fairly well summarized. There’s some attempt to elucidate the differences and similarities between their study and other quoted studies. Under the study strengths, you state that "the study site is a National Referral and Teaching hospital whose staff are likely to have a lot of influence elsewhere within the country". Is it the national referral status that makes the impact influence or the stakeholders you interviewed, including policy makers who have more influence.

Thank you so much for the comment. You are right. Most likely the policy makers have more influence. Most of study participants in this research who are at policy level and National Referral/Teaching Hospital are people who commonly participate in support supervision, training and mentorships to improve reproductive, maternal, newborn and adolescent health (RMNCAH) related services including efforts to strengthen maternal and perinatal death surveillance and response (MPDSR) in other parts of the country. Many of them again participate in policy discussions. Therefore, both groups (at policy level and National Referral/Teaching Hospital) are likely to learn from and use our findings to address barriers and scale up maternal death surveillance and response implementation in the rest of the country.   

# Peer review: Reviewer response for version 1

**DOI:** 10.21956/openresafrica.14587.r29318

**Published:** 2022-10-19

**Authors:** Wendy J. Graham

**Affiliations:** 1 Department of Infectious Disease Epidemiology, London School of Hygiene and Tropical Medicine, London, UK

This is an interesting paper with the potential to make an important contribution to on-going efforts to strengthen the implementation of MPDSR* across low-&-middle-income countries. The application of the Theory of Planned Behaviour to guide this indepth research is novel and helpful, and the authors are to be congratulated on the thoroughness of their interpretation of the qualitative data.

 There is, however, one major concern with the paper which requires the authors’ action. As noted through out the paper, given the sensitivity of the tragedy of preventable maternal deaths and the real risks of inappropriate blame, indeed extending into legal redress, those taking part in this type of research – especially at one named hospital, need to have additional safeguards around confidentiality and anonymity. Although the authors describe good practices around informed consent, they should be requested to offer the journal further reassurance that the participants, listed in Table 1, cannot be identified. There is, for example, one anesthetist who participated, and readers of the paper familiar with this hospital maybe able to gauge who this is, and the same for the other senior clinicians. Similarly, for the external stakeholders, the participating UN organizations are mentioned and the paper indicates those taking part knew about MDSR, so again it may well be possible in Uganda to identify the specific individuals. Although it is widely-regarded as good practice to give direct quotations and attribute these by type of participant, some of the statements – especially on blame on p.8 & 10 – are sensitive (e.g. “respondent laughs”), and again – extra care is needed to maintain anonymity.

 One way round this sort of confidentiality challenge is not to name the participating hospital, and it would be good for the authors to consider this, especially as some of the critique of managers is quite explicit, and may have consequences for their future willingness to participate in research. Of course, this is not suggesting that findings should be changed or toned-down in any way, but from a research perspective and the journal audience, the name of the hospital is not relevant but rather its level or characteristics. There is a separate matter of feeding back findings to the participating hospital, when again the confidentiality of participants needs to be protected, but decision-makers and managers certainly could benefit from the study in terms of local improvements. Once again, it is important for the authors to reflect on this important issue, and for the journal to receive a response prior to indexing.

 There are a few other suggestions which could improve the paper:

1. Stakeholder selection – more detail would be helpful on how these individuals were chosen, and particularly the rationale for including those with no prior training in MDSR. With nearly half of the sample (43%) being untrained, this makes some of the questions about the purpose and processes of MDSR hard for them to answer. In fact, a case could be made for separating out the responses of those trained from those untrained, and this could yield more meaningful findings and probably some useful comparisons. Indeed, given the huge diversity of participants, reporting how the findings varied across these different constituencies would be useful throughout.
2. Position of lead researcher at the participating hospital. It is good to read of the partnership between a clinical and social scientist for the conduct of the study, but for the former – it is important to know if they are also a clinician at the facility and if so, how this may have impacted on the conduct of research and the findings.
3. Medical causes of maternal deaths – on p.5 it mentions perceptions of the main causes according to participants, but it would be helpful to compare this with what the MDSR itself reports as the main causes.
4. Discussion & Conclusion - this is well-structured and informative, but concluding with a long list of barriers and facilitators without any suggestions on prioritization is a missed opportunity. Many of these elements have been noted in other studies of MDSR – especially around the lack of action step, HR shortages, lack of training and the importance of leadership, so are not new contributions to the field. The unique addition comes from the authors’ use of their local understanding and insights to make valid suggestions on priorities.

**Minor comments: **

1. MPDSR or MDSR – it would be helpful to clarify at the beginning of the paper why this study is just on maternal deaths, and whether Uganda is also conducting MPDSR. The paper seems to use both abbreviations synonymously.
2. Some editorial improvements would be helpful as some sentences are not clear and seemingly some words or punctuation is missing e.g. last sentence of 2 ^nd^ paragraph.

Is the work clearly and accurately presented and does it cite the current literature?

Yes

If applicable, is the statistical analysis and its interpretation appropriate?

Yes

Are all the source data underlying the results available to ensure full reproducibility?

Yes

Is the study design appropriate and is the work technically sound?

Partly

Are the conclusions drawn adequately supported by the results?

Partly

Are sufficient details of methods and analysis provided to allow replication by others?

Yes

Reviewer Expertise:

I am an obstetric epidemiologist, with 30+ years of experience in the measurement of maternal mortality, including the use of MDSR, and have worked in partnership with colleagues in many low and middle-income countries.

I confirm that I have read this submission and believe that I have an appropriate level of expertise to confirm that it is of an acceptable scientific standard, however I have significant reservations, as outlined above.

# Response

**Published:** 2023-03-17

**Authors:** Imelda Namagembe

**Affiliations:** Obstetrics and Gynaecology, Makerere University, College of Health Sciences / Mulago National Referral Hospital, Kampala, Uganda, Uganda

**Response to reviewer report from Wendy J. Graham** Department of Infectious Disease Epidemiology, London School of Hygiene and Tropical Medicine, London, UK

 This is an interesting paper with the potential to make an important contribution to on-going efforts to strengthen the implementation of maternal and perinatal death surveillance and response (MPDSR) across low-&-middle-income countries. The application of the Theory of Planned Behaviour to guide this in-depth research is novel and helpful, and the authors are to be congratulated on the thoroughness of their interpretation of the qualitative data.

**Response: **Thank you so much for the encouraging and kind note about our paper.

 There is, however, one major concern with the paper which requires the authors’ action. As noted throughout the paper, given the sensitivity of the tragedy of preventable maternal deaths and the real risks of inappropriate blame, indeed extending into legal redress, those taking part in this type of research – especially at one named hospital, need to have additional safeguards around confidentiality and anonymity. Although the authors describe good practices around informed consent, they should be requested to offer the journal further reassurance that the participants, listed in Table 1, cannot be identified. There is, for example, one anesthetist who participated, and readers of the paper familiar with this hospital may be able to gauge who this is, and the same for the other senior clinicians.

**Response:** Thank you so much for concern related to how to strengthen safeguards around confidentiality of information from this major study site to minimize risk of inappropriate blame to study participants. It is highly unlikely to identify the study participants individually. A group of people can be thought of having participated in the research, but not individual identification because the specific positions of the study participants have more than one person. In addition, even facility leaders or external stakeholders’ voices are referred have been referred to as “ ***leaders/managers”*** when it came to quotes. In addition, source of the voices f(quotes) of study participants have been edited in such a manner that reflects a specific cadre not individual name such as midwife, enrolled midwives, registered midwives, OBS-GYN (Specialist-Obstetrician-Gynaecologist), senior house officers (SHO) or support staff. Leaders/Managers representing all forms of leaders (facility leaders, external stakeholders from Ministry of Health or other Implementing partners).  For quotes where we had put e.g “facility leader” or “ministry of health person” has been changed to “Leader/managers”. Where we had one anaesthesia provider, has been changed to “anaesthesia providers”. Two anaesthesia providers participated in the interviews, the 3 ^rd^ one failed to create time from the busy schedules and the 4 ^th^ one was called for an emergency at the start of a focus group discussion. We had 15 anaesthesia providers at the facility by then (anaesthetic officers & anaesthesiologists) . We believe that it is really hard to point out who of those 15 participated in the research. available participated. The study participants themselves and research team are the only people who are aware of those involved.

 Similarly, for the external stakeholders, the participating UN organizations are mentioned and the paper indicates those taking part knew about MDSR, so again it may well be possible in Uganda to identify the specific individuals. Although it is widely regarded as good practice to give direct quotations and attribute these by type of participant, some of the statements – especially on blame on p.8 & 10 – are sensitive (e.g. “respondent laughs”),

**Response: **Thank you so much for raising this comment again to strengthen confidentiality. The phrase “respondent laughs” has been deleted from that quote on page 8 without losing meaning”. Current quote is ‘..sometimes we try to point fingers and even blame colleagues...(Resp-1-Midwife, IDI).  We had not considered the end phrase “respondent laughs” to be so sensitive. This aspect has been rectified. Although the names of the participating organizations (for external stakeholders) have been mentioned to enhance authenticity of the source of information given, the phrase “representatives from the implementing partners has been added” (under the methods section where we talk about other participants). At the time of reflecting the voice of external stakeholder / facility leader / administrator/ head of department we have instead used “Leaders/Managers” as source of that voice. We are of the opinion that this does not point to a particular organization as source of that voice or a specific leader but a group of people who fall in that category.

 and again – extra care is needed to maintain anonymity. One way round this sort of confidentiality challenge is not to name the participating hospital, and it would be good for the authors to consider this, especially as some of the critique of managers is quite explicit, and may have consequences for their future willingness to participate in research. Of course, this is not suggesting that findings should be changed or toned-down in any way, but from a research perspective and the journal audience, the name of the hospital is not relevant but rather its level or characteristics.

**Response:** Thank you so much for raising this concern again to enhance confidentiality. In this paper, we commonly refer to the main study site as “a busy National Referral Hospital”. Although it may be hard to anonymize the Hospital completely, we have removed the name and other descriptions likely to identify the hospital. What is encouraging is that at the moment, there is ongoing nationwide call to strengthen maternal and perinatal death surveillance and response (MPDSR) to reduce deaths ***.*** There is emphasis to minimize blame while enhancing responsibility and accountability. The National Referral Hospital has been prioritized by Ministry of Health and partners with the purpose of supporting it in view of its high volume of deliveries (> 20,000 per year), the training role and high burden of preventable maternal and perinatal deaths. There are efforts to streamline referrals to this setting, transform it into a Centre of excellence in a number of areas including strengthening maternal and perinatal death surveillance and response (MPDSR) in general. We believe this will further reduce the sensitivity around maternal death notification and review. In addition, the advice from experts from National Referral Hospital and University institutions is greatly respected since they are considered as the mentors to strengthen uptake of quality improvement activities. In 2020, the “National Safe Motherhood Expert Committee (NASMEC) was created, it has experts from across the country including National Referral Hospital. The NASMEC works closely with the Ministry of Health and Implementing partners to scale up a number of quality improvement activities including MPDSR.  Of late, the Ministry of health, national MPDSR committee, NASMEC and health facility leaders from various regions of the country freely discuss MPDSR issues on widely shared Media platforms, and lessons learnt are shared to improve outcomes.

 There is a separate matter of feeding back findings to the participating hospital, when again the confidentiality of participants needs to be protected, but decision-makers and managers certainly could benefit from the study in terms of local improvements. Once again, it is important for the authors to reflect on this important issue, and for the journal to receive a response prior to indexing.

**Response: **Thank you so much for this comment and raising the fact that it is important to give feedback to the national referral site. Since this was an implementation research project with a before and after component, feedback has been given and discussions to improve implementation of MPDSR have been ongoing without disclosing details of study participants. During the feedback meetings, participants freely discussed the findings focusing on what can be done to improve implementation. Research related engagement and feedback has been ongoing to share findings and lessons from local data.  There has been no disclosure of individual data sources but reported as grouped data e.g the midwives reported that or the seniors house officers or the .leaders/managers  raised such a concern. The aspect of utilizing local data to strengthen implementation of the policy on maternal and perinatal death surveillance and response to inform quality improvement activities as part of the “Response” as a whole has been greatly appreciated.

 There are a few other suggestions which could improve the paper: Stakeholder selection – more detail would be helpful on how these individuals were chosen, and particularly the rationale for including those with no prior training in MDSR. With nearly half of the sample (43%) being untrained, this makes some of the questions about the purpose and processes of MDSR hard for them to answer. In fact, a case could be made for separating out the responses of those trained from those untrained, and this could yield more meaningful findings and probably some useful comparisons. Indeed, given the huge diversity of participants, reporting how the findings varied across these different constituencies would be useful throughout.

**Response: Thank you so much for the comment.** **The stakeholders’ selection was as follows**: The inclusion criteria for the study participants for IDIs, FGDs for the health workers or KIIs for leaders/managers at the National Referral Hospital or external stakeholders required having stayed at the respective position for at least six months. For both the internal and external stakeholders, a preliminary list was developed in conjunction with officials at the National Referral Hospital and the Ministry of Health. The initial study participants also recommended others who they thought would enrich understanding of the barriers and facilitators of MDSR implementation. The identified people were then contacted by one of the research team members or Principal investigator (NI), first author, informed about the research and requested to give an appointment to participate. Ten out of the 77 potential participants contacted failed to create time because of their busy schedule (3-SHOs, 3-midwives and two anaesthesia providers plus some two of the external stakeholders (from Ministry of Health). The fact that some participants excused themselves (did not participate) gave us some level of confidence that participants decision was voluntary. .A written consent was obtained before actual participation. The study participants were informed earlier that their input would greatly inform what should be done to strengthen implementation of the maternal death surveillance and response policy as a quality improvement process since the retrospective quantitative maternal death- data had shown that the proportion of deaths reviewed was still low (at 33 %) and not done in a timely manner (Namagembe et al., 2022). Exposure to previous training in maternal or perinatal death surveillance and response was not used as criteria to select study participants since some messages to support MPDSR activities used to be discussed in departmental meetings in a non-selective manner even before this research. However, during collection of biodata, a question was included to have an idea of those that had previous exposure to formal training in MPDSR processes. When we explored the data qualitatively, there were no differences regarding the identified barriers or facilitators based on whether someone had formal training in MPDSR previously or not.

**Other aspects **

1. Position of lead researcher at the participating hospital. It is good to read of the partnership between a clinical and social scientist for the conduct of the study, but for the former – it is important to know if they are also a clinician at the facility and if so, how this may have impacted on the conduct of research and the findings.

**Response: **Thank you for raising these comments and thank you for appreciating the partnership with the social scientists (MM) during this qualitative research. **Reflexivity concerns:** Although NI is one of the Senior Clinicians at the study hospital, she made read more and also made enquiries from social scientists (senior qualitative researchers) who proposed that she can participate as one of the great tools in this research. However, she continuously self-examined and remained aware of the need to detach self from the phenomenon being explored in this case MDSR in order to capture participants’ views with an open mind. This involved remaining neutral and setting aside views of the ‘clinician’ to fit in the role of a researcher guided by study objectives and a pre-designed interview guide with clear topics for discussion during the conduct of interviews. In addition, the lead researcher was commonly introduced ‘as senior-midwife’ in order to minimize positionality to a certain degree to enhance a free environment of communication. NI put in a deliberate effort work with independent social scientist and note takers and participants were encouraged to give their correct perspectives. No participant was addressed by name during interviews. It is true that in reflexivity, some researchers tend to carry their own perspectives during the conduct of research and interpretation of findings. This challenge was mitigated by working with an independent social scientist, using open ended questions, having an independent note taker who did the initial transcription and independent qualitative researchers who worked on the initial coding of the data and later discussed with NI and other co-authors. The variety of health workers in the study plus use of IDIs, KIIs and FGDs helped to enhance triangulation of data. The nature of the interviews looked at both barriers and facilitators to get a more balanced enquiry. Therefore, we feel that these efforts enhanced trustworthiness of the information presented in this research. As recommended by some researchers (Lincoln and Guba, 1986) and (Connely Lynne M, 2016), there was effort to enhance credibility, confirmability and dependability.

1. Medical causes of maternal deaths – on p.5 it mentions perceptions of the main causes according to participants, but it would be helpful to compare this with what the MDSR itself reports as the main causes.

**Response:** Thank you so much for this comment. It is important to note that the causes of death reported by majority of the participants’ during the qualitative study do agree with the findings from quantitative maternal death data (excessive bleeding due to postpartum hemorrhage as number 1, followed by complications of hypertensive disorders, puerperal infection, abortions related complications and others (Namagembe et al, 2022; Kaye et al, 2003). However, other aspects such as negative attitude of some of the health workers and under table payments were newly identified in the qualitative study.

1. Discussion & Conclusion - this is well-structured and informative, but concluding with a long list of barriers and facilitators without any suggestions on prioritization is a missed opportunity. Many of these elements have been noted in other studies of MDSR – especially around the lack of action step, HR shortages, lack of training and the importance of leadership, so are not new contributions to the field. The unique addition comes from the authors’ use of their local understanding and insights to make valid suggestions on priorities.

**Response: Thank you so much for this comment. Most of the participants suggested the following priority interventions**

- To prioritize training of all health workers including midwives and administrators in both maternal and perinatal death surveillance and response (MPDSR)
- To prioritize capacity building to strengthen health workers’ skills in emergency care to prevent complications and manage patients in a timely manner in case complications occur.
- Discuss modalities to eliminate blame from leaders/ managers and specialists, but strengthen accountability and endeavor to streamline the legal framework for MDSR.
- Strengthen leadership and governance at health facility and national levels to ensure continued supervision of health workers, timely referral and feedback
- Strengthen regular engagement of various stakeholders to enhance implementation of recommendations from MDSR. 
- Increase funding: Lobby the Ministry of Health, Parliament Ministry of finance, and partners to allocate adequate resources for MDSR implementation and address the challenges of patient overload, availability of emergency supplies including blood, at the National Referral Hospital and functionalize referring facilities.

1. Minor comments: MPDSR or MDSR – it would be helpful to clarify at the beginning of the paper why this study is just on maternal deaths, and whether Uganda is also conducting MPDSR. The paper seems to use both abbreviations synonymously.

**Response: Thank you so much for raising this concern. ** True, Uganda is advocating for both maternal and perinatal death surveillance and response (MPDSR). The principal investigator had an original plan to conduct research on both aspects comprehensively as MPDSR. but, she was advised that as an obstetrician, it was better to focus on the maternal component since it was not being done as recommended by Ministry of Health. This happened at both the Departmental level and Research/ Ethics and clearance committee. However, during the training intervention, stakeholder engagement sessions and actual application of the practice, both maternal and perinatal death surveillance components were emphasized. The discussions at the facility level have shown improvement for both maternal and perinatal death notifications and reviews post intervention.

1. Some editorial improvements would be helpful as some sentences are not clear and seemingly some words or punctuation is missing e.g. last sentence of 2nd paragraph.

**Response: Thank you so much for the comment**. The last paragraph has been edited and changed to read follows: “There is limited information on barriers and facilitators to timely MDSR in high-volume settings in SSA where MDSR uptake is still low”        
